# *Atractylodes lancea* Rhizome Polysaccharide Alleviates MCD Diet-Induced NASH by Inhibiting the p53/mTOR Pathway

**DOI:** 10.3390/ijms252011112

**Published:** 2024-10-16

**Authors:** Dajin Pi, Zheng Liang, Maoxing Pan, Jianwei Zhen, Chuiyang Zheng, Jinyue Pan, Wen Fan, Qingliang Song, Qinhe Yang, Yupei Zhang

**Affiliations:** School of Traditional Chinese Medicine, Jinan University, Guangzhou 510632, China

**Keywords:** *Atractylodes lancea* rhizome polysaccharide, p53/mTOR, non-alcoholic steatohepatitis

## Abstract

Nonalcoholic steatohepatitis (NASH) is a form of chronic liver disease that is characterized by liver inflammation and steatosis, with possible progression to fibrosis. Currently, no drugs have been approved for the treatment of NASH. In this study, we isolated a polysaccharide from *Atractylodes lancea* rhizome (AP) and established a methionine- and choline-deficient (MCD) diet -induced NASH mouse model to investigate the preventive effect and potential mechanism of AP on NASH. The results showed that AP effectively reduced liver lipid accumulation and inflammation and reduced autophagy and ferroptosis in hepatocytes, thereby preventing the development of NASH. These findings suggest that AP may be a promising natural candidate for the treatment of NASH.

## 1. Introduction

Nonalcoholic steatohepatitis (NASH) is a clinicopathological syndrome characterized by hepatocyte steatosis and inflammation in the absence of alcohol or other definite factors [1]. NASH is a critical stage in the progression of nonalcoholic fatty liver disease (NAFLD). Without intervention, NASH can progress to fibrosis, cirrhosis, and even liver cancer [2]. Studies have shown that the prevalence of NASH in the global population ranges from 1.5% to 6.5% and that the prevalence is projected to increase by 63% by 2030 [3]. The pathogenesis of NASH is not very clear and may be related to oxidative stress, the inflammatory response, mitochondrial damage, and intestinal flora disorders [4]. The recommended treatment for NASH remains limited to lifestyle modifications [5]. Therefore, exploring natural drugs for the prevention and treatment of NASH without side effects has become an urgent endeavor.

*Atractylodes lancea* rhizome is a well-known herb in East Asia that has been used clinically for thousands of years [6]. It mainly contains a variety of chemical components, such as sesquiterpenoids, monoterpenes, polyacetylenes, phenolic acids, and steroids; the extracts and chemical components have been proved to have effective hepatoprotective effects [7]. Atractylodin is the main active component of *Atractylodes lancea* rhizome and studies have shown that atractylodin can alleviate high-fat diet-induced NAFLD by inhibiting hepatocyte ferroptosis [8]. Studies have shown that *Atractylodes lancea* rhizome polysaccharide can alleviate intestinal flora imbalance and short chain fatty acids (SCFAs) metabolic disorder by protecting the intestinal mucosal barrier, and at the same time, strengthen the intestinal mucosal immunity [9]. In recent years, with the in-depth study of polysaccharides, it has been found that polysaccharides have a variety of biological effects, such as anti-aging, antioxidative, anti-inflammatory, liver protection, hypoglycemic, lipid-lowering, antiviral, antitumor, and immune regulation effects, and improve glucose metabolism disorders [10,11,12,13]. Therefore, they have attracted increasing attention.

In this study, we isolated a polysaccharide from *Atractylodes lancea* rhizome (AP) and used a methionine- and choline-deficient (MCD) diet-induced mouse model to evaluate its efficacy in the treatment of NASH. Hematoxylin-eosin (HE) staining and Oil Red O staining were used to evaluate the degree of fatty liver, biochemical kits were used to measure blood lipids and assess liver function, lipidomics and transcriptome sequencing were used to identify the molecular mechanisms of lipid changes and drug effects in mice, and Western blotting was used to analyze the expression of autophagy-related and ferroptosis-related proteins in liver tissue. Autophagy inhibitors were used to explore the relationship between autophagy and ferroptosis to systematically study the preventive and therapeutic effects and mechanism of AP on NASH.

## 2. Results

### 2.1. Structural Analysis of AP

**Figure 1:**
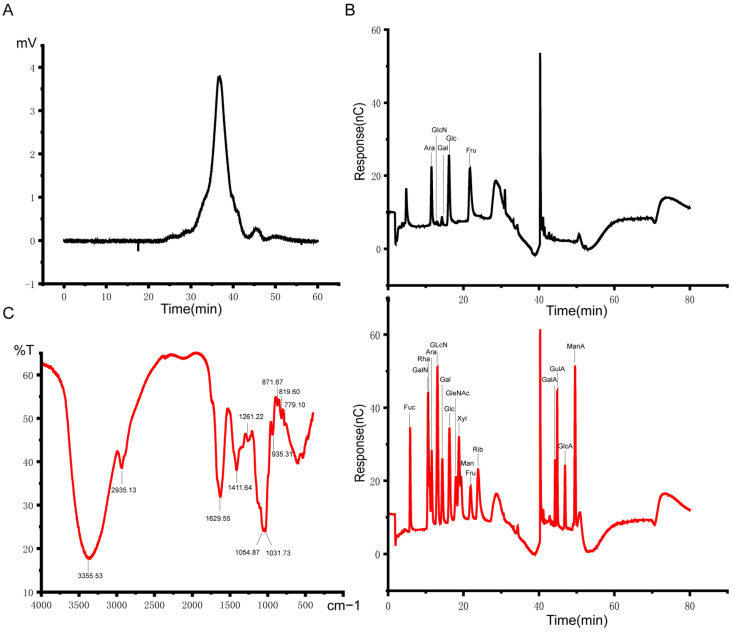
Structural analysis of AP. (**A**) Molecular weight of AP. (**B**) Monosaccharide composition of AP. (**C**) FT-IR spectra.

High-performence gel permeation chromatography(HPGPC) analysis of AP samples revealed four peaks; the average molecular weights of the four peaks were 543966 Da, 63397 Da, 11969 Da, and 3246 Da, and the retention times were 25.757 min, 30.333 min, 33.882 min, and 36.660 min (Figure 1A). The monosaccharide composition analysis showed that AP had five single peaks, corresponding to arabinose, glucosamine hydrochloride, galactose, glucose, and fructose with a molar ratio of 93:2:16:106:784. These results indicate that AP consists of a polymer of five monosaccharides (Figure 1B). FT-IR analysis can reveal different functional groups and facilitate the analysis of different characteristic structures. Absorption bands at 3600~3200 cm^−1^ indicate the stretching vibration absorption peak of -OH; absorption peaks in this region are characteristic of polysaccharides. In this study, the peak at 3355 cm^−1^ was the stretching vibration absorption peak of O-H, a characteristic peak of saccharides. There was an absorption peak at 2935 cm^−1^, potentially attributed to the C-H stretching vibration. There was an absorption peak at 1629 cm^−1^, potentially attributed to the C=O asymmetric stretching vibration. There was an absorption peak at 1411 cm^−1^, potentially attributed to the C-O stretching vibration. The absorption peaks at 1261 cm^−1^, 1054 cm^−1^, and 1031 cm^−1^ may be attributed to O-H variable angle vibrations. There was an absorption peak at 935 cm^−1^, potentially attributed to the asymmetric ring stretching vibration. There was an absorption peak at 871 cm^−1^, potentially attributed to the C-H variable angle vibration of the equatorial bond other than the end-base C-H epimer. There was an absorption peak at 819 cm^−1^, potentially attributed to the C-H variable angle vibration of the α-terminal group epimorphism. There was an absorption peak at 779 cm^−1^, potentially attributed to the symmetric ring stretching vibration (Figure 1C).

### 2.2. AP Improved Hepatic Steatosis and Hepatocyte Injury

**Figure 2:**
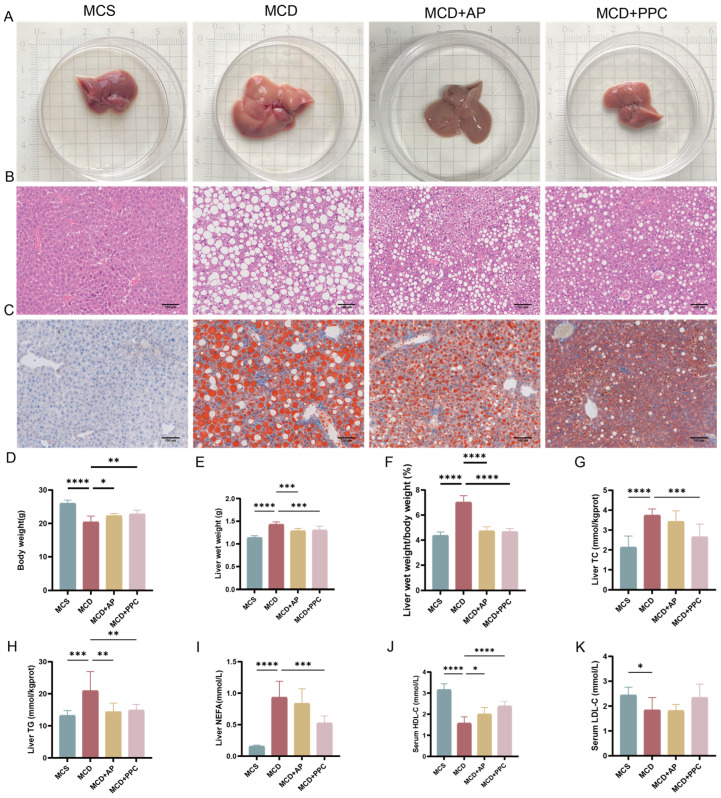
AP had therapeutic effects in the NASH model. (**A**) Macroscopic observation of the livers. (**B**,**C**) Representative images of HE staining of liver paraffin sections, Oil Red O staining of frozen liver slides observed under a microscope (scale bar: 100 µm; original magnification, ×200). (**D**–**F**) Body weight, liver wet weight, and liver wet weight/body weight of mice in each group. (**G**–**I**) TC, TG, and NEFA contents in the livers of mice in each group. (**J**,**K**) Serum concentrations of HDL-C and LDL-C. * *p* < 0.05; ** *p* < 0.01; *** *p* < 0.001; **** *p* < 0.0001.

Liver tissue HE staining showed that the liver of mice in the MCD group had extensive steatosis, and Oil Red O staining showed an increase in lipid droplets in the liver of mice in the MCD group. However, these conditions were improved after AP and PPC treatment (Figure 2A–C). The MCD group lost body weight, while AP and PPC treatment restored body weight (Figure 2D). Liver wet weight and liver wet weight/body weight increased in mice in the MCD group, indicating liver lipid accumulation. However, AP treatment reduced liver weight, suggesting that AP and PPC decreased lipid accumulation in the livers of mice (Figure 2E,F). TG, TC, and NEFA levels were increased in MCD mice; however, AP and PPC treatment reduced liver TG, TC, and NEFA levels, suggesting that AP can normalize lipid metabolism (Figure 2G–I). However, at the same time, AP treatment improved HDL-C levels but had no effect on LDL-C levels. (Figure 2J,K).

### 2.3. AP Extensively Modulated the Hepatic Lipid Profile

**Figure 3:**
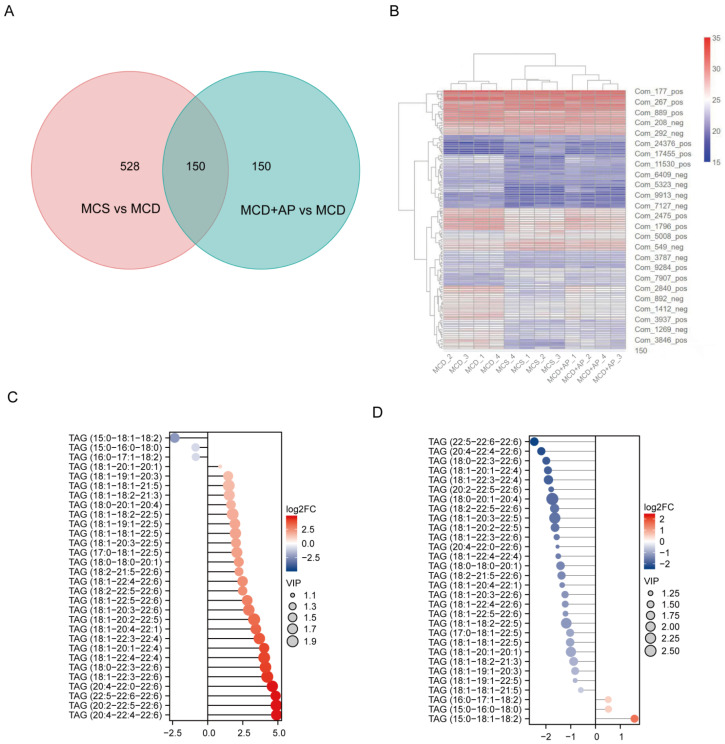
AP broadly modulates hepatic lipid metabolism. Lipidomic analysis was conducted on mouse liver tissue samples (*n* = 4). (**A**) A Venn diagram was created for the DALs. (**B**) DALs were clustered to create a heatmap. The *x*-axis denotes sample names and hierarchical clustering results, while the *y*-axis represents DALs and their hierarchical clustering results. (**C**) Matchstick plot of DALs for the MCD group and MCS group. (**D**) Matchstick plot of DALs for MCD+AP in comparison to the MCD group. The *x*-axis denotes log2 fold change, while the *y*-axis represents differential TAG. VIP: Variable Importance in Projection.

To further verify the effect of AP on liver lipid metabolism in fatty liver, lipidomic analysis was used to assess the changes in the liver lipid profile in the MCS group, MCD group, and MCD+AP group. Through lipidomic analysis, we screened 150 lipid metabolites that were differentially altered among the MCS group, the MCD group, and the MCD+AP group (Figure 3A). After performing cluster analysis on these differential lipids, it was found that the differential lipids in the MCS, MCD+AP, and MCD groups showed a distinct hierarchical clustering pattern (Figure 3B).

It is well known that triacylglycerol (TAG) is the main form of fat accumulation in the liver and is closely associated with NASH. Therefore, by comparing the expression of the top 30 TAGs between pairs of groups, we found that, compared to the MCS group, the levels of liver lipid TAGs such as TAG (18:1-20:1-20:1), TAG (18:1-19:1-20:3), and TAG (18:1-18:1-21:5) in the MCD group were significantly increased (Figure 3C). After AP intervention, the treatment group significantly reversed the upward trend of TAGs compared to the MCD group (Figure 3D).

These findings suggest that AP may exert protective effects against NASH, at least in part, by modulating the hepatic lipid profile and lipid accumulation. This extensive modulation of liver lipids contributes to the observed normalization in liver histology and function, further supporting the potential therapeutic benefit of AP in the treatment of NASH.

### 2.4. AP Alleviated Hepatocyte Inflammation Associated with NASH

**Figure 4:**
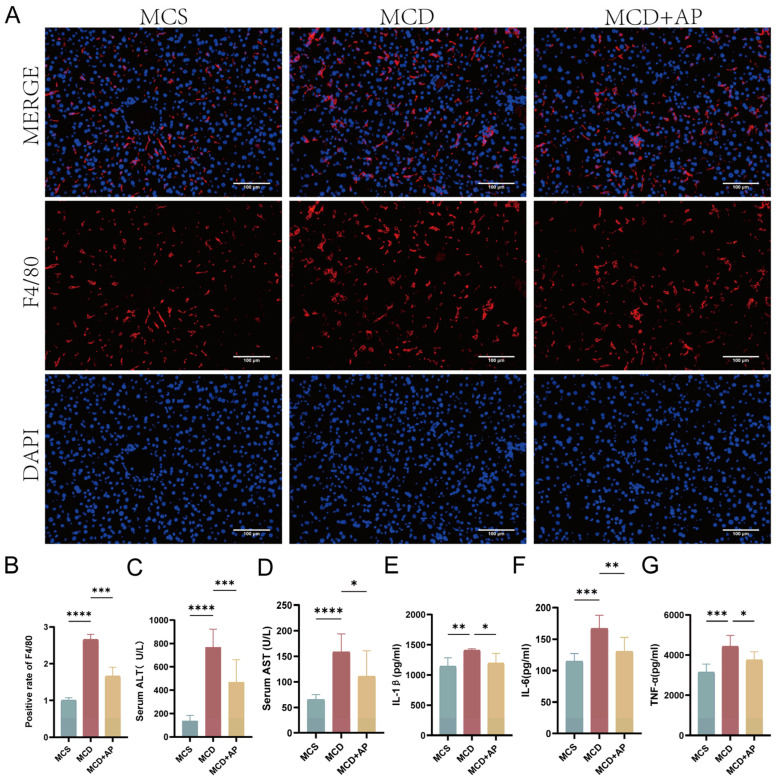
The development of steatohepatitis in mice fed an MCD diet can be reduced by treatment with AP. (**A**) Micrographs of F4/80 staining in hepatic sections (scale bar, 100 µm; 200× original magnification). (**B**) Positive rate of F4/80 (*n* = 3). (**C**,**D**) Serum AST and ALT levels. The findings are presented as the average ± standard deviation (*n* = 6). (**E**–**G**) Levels of liver IL-1*β*, IL-6, and TNF-*α*. The findings are presented as the mean ± standard deviation (*n* = 6). * *p* < 0.05; ** *p* < 0.01; *** *p* < 0.001; **** *p* < 0.0001.

AP treatment can reduce the progression of steatohepatitis in NASH model mice. Immunofluorescence (IF) staining results showed that the fluorescence intensity of F4/80 was the highest in the livers of mice in the MCD group and that the increase in liver macrophage infiltration in NASH mice after AP treatment was reduced, indicating that AP treatment reduced macrophage recruitment to the liver (Figure 4A,B). Serum AST and ALT levels and liver TNF-*α*, IL-6, and IL-1*β* levels were increased in the MCD group; AP treatment reversed this trend (Figure 4C–G).

### 2.5. AP Regulates the Expression of Autophagy and Ferroptosis-Related Genes Through p53

**Figure 5:**
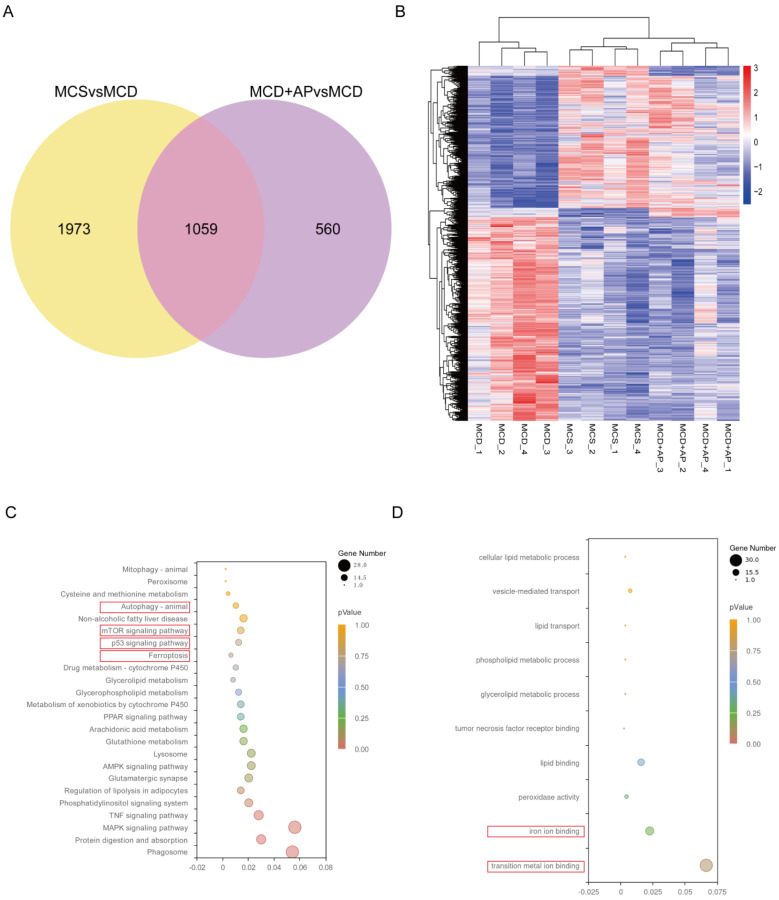
AP extensively regulates the liver transcriptome. RNA-seq analysis was conducted on mouse liver tissue samples (*n* = 4). (**A**) A Venn diagram was created for the DEGs. (**B**) DEGs were clustered to create a heatmap. The *x*-axis denotes sample names and hierarchical clustering results, while the *y*-axis represents DEGs and their hierarchical clustering results. Red indicates high expression, and blue indicates low expression. (**C**) KEGG analyses of the DEGs. The *y*-axis shows KEGG pathways, and the *x*-axis represents the Rich factor. A larger Rich factor indicates a higher degree of enrichment. Larger dots signify a greater number of DEGs enriched in the pathway, and redder dots indicate more significant enrichment. (**D**) Enriched GO terms. The *y*-axis shows GO pathways, and the *x*-axis represents the Rich factor. The red square frames represent key biological processes and pathways in this study.

We used RNA sequencing (RNA-seq) to reveal the underlying mechanism by which AP alleviates NASH. First, by separately screening the differentially expressed genes (DEGs) in the livers of mice from the MCS group, MCD group, and MCD+AP group, we identified 1059 common DEGs by taking the intersection of these genes (Figure 5A). After performing clustering analysis on these DEGs, it was found that the differential genes in the MCS, MCD+AP, and MCD groups exhibited a clear hierarchical clustering pattern, with gene expression in the MCD+AP group being more similar to that in the MCS group, indicating that the gene expression of the treatment group was closer to the normal group (Figure 5B). KEGG pathway analysis identified multiple enriched pathways, including those related to lipid metabolism, inflammation, oxidative stress, p53, mTOR, autophagy, and ferroptosis (Figure 5C). According to GO-Biological Process (GO: BP) analysis, the DEGs were enriched in lipid metabolism and the biological processes involved in iron ion binding in lipid metabolism (Figure 5D). These results suggest that AP can play a protective role in NASH by participating in the regulation of gene expression related to key biological processes and pathways such as autophagy and ferroptosis.

### 2.6. AP Inhibits Hepatic Autophagy

**Figure 6:**
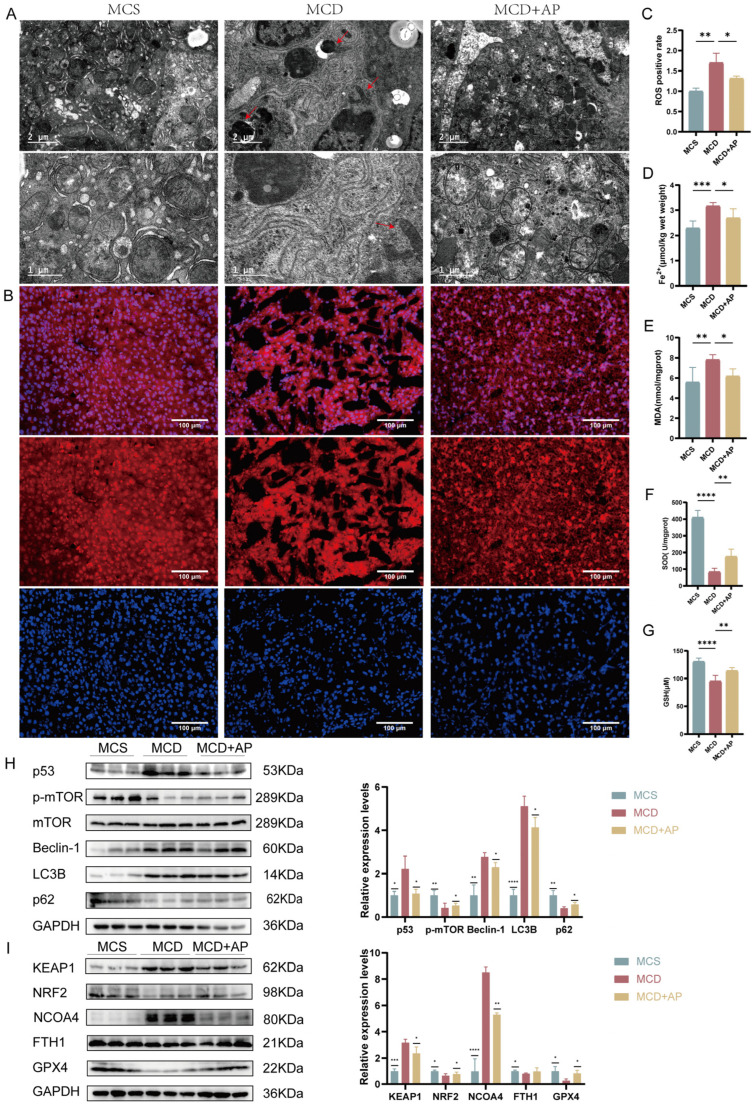
The progression of autophagy and ferroptosis in MCD-fed mice can be mitigated with AP treatment. (**A**) Ultrathin liver sections observed under TEM (7200× and 23,000×). (**B**,**C**) ROS staining and relative expression levels (scale bar: 100 µm; 200×). (**D**–**G**) Liver Fe^2+^, MDA, SOD, and GSH levels (*n* = 6). (**H**,**I**) The relative protein expression levels of p53, *p*-mTOR, Beclin-1, LC3B, p62, KEAP1, NRF2, NCOA4, FTH1, and GPX4 were assessed by Western blot analysis. The data are presented as the mean ± standard deviation. Red arrows represent typical organelle structures of ferroptosis or autophagy. * *p* < 0.05; ** *p* < 0.01; *** *p* < 0.001; **** *p* < 0.0001.

To further investigate the effect of AP on hepatocyte autophagy in NASH mice, we used TEM to observe the structure of hepatocytes and Western blotting to analyze the expression of autophagy-related proteins. TEM results showed that the mitochondria in the MCD group were surrounded by membrane structures, indicating the occurrence of mitophagy. However, AP treatment improved mitophagy (Figure 6A). As shown in Figure 6H, we further verified the above results by analyzing the protein expression of p53, *p*-mTOR, Beclin-1, LC3B, and p62. In the MCD group, we observed increased expression levels of p53, Beclin-1, and LC3B, and decreased expression levels of *p*-mTOR and p62, indicating enhanced autophagy. After AP treatment, the expression levels of p53, Beclin-1, and LC3B decreased, and the expression levels of p-mTOR and p62 increased, indicating that autophagy decreased. These results suggest that AP may play a protective role against NASH by regulating the p53/mTOR pathway to improve impaired autophagy in hepatocytes.

### 2.7. AP Reduces NASH by Inhibiting Ferroptosis

The results of TEM showed that the volume of mitochondria in the liver of the MCD group was reduced, accompanied by breakage and loss of cristae, indicating that ferroptosis occurred in the MCD group. However, AP treatment reversed the mitochondrial damage. To explore the potential role of ferroptosis in the protective mechanism of AP against NASH, we assessed the immunofluorescence levels of ROS and the concentration of Fe^2+^, which are closely related to ferroptosis (Figure 6B–D). The immunofluorescence results showed that ROS levels in the hepatocytes of mice in the MCD group were increased, suggesting oxidative stress and potential ferroptosis. However, AP treatment reduced ROS levels. We confirmed this result by analyzing the levels of MDA, SOD, and GSH (Figure 6E–G). In the mice in the MCD group, we observed increased MDA activity and decreased SOD and GSH activities. However, AP treatment decreased MDA activity and increased SOD and GSH activities, indicating that AP enhanced the antioxidant capacity of hepatocytes, thereby inhibiting ferroptosis. Finally, we analyzed the expression of five key regulators of ferroptosis, KEAP1, NRF2, NCOA4, FTH1, and GPX4, by Western blotting (Figure 6H). We observed increased KEAP1 and NCOA4 expression and decreased NRF2, FTH1, and GPX4 expression in the MCD group, suggesting potential ferroptosis. The expression of KEAP1 and NCOA4 decreased after AP treatment. NRF2, FTH1, and GPX4 increased, further confirming the inhibition of ferroptosis. These results suggest that AP may exert its protective effect on NASH by inhibiting ferroptosis.

### 2.8. Intervention with 3-MA Validated That Autophagy Precedes Ferroptosis

**Figure 7:**
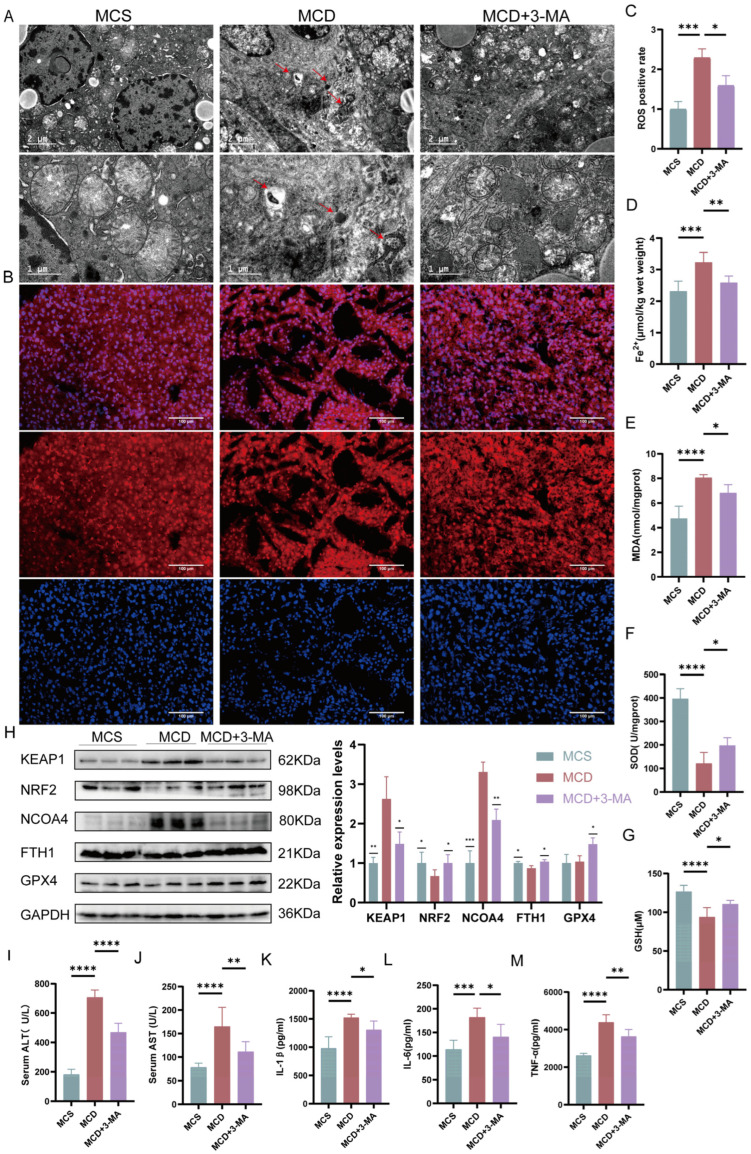
3-MA had therapeutic effects in a NASH mouse model. (**A**) Liver ultrathin sections observed under TEM (7200× and 23,000×). (**B**,**C**) ROS staining and relative expression levels (scale bar: 100 µm; 200×). (**D**–**G**) Liver Fe^2+^, MDA, SOD, and GSH levels (*n* = 6). (**H**) The relative protein expression levels of KEAP1, NRF2, NCOA4, FTH1, and GPX4 were verified by Western blot analysis. (**I**,**J**) Serum AST and ALT levels. (**K**–**M**) Liver IL-1*β*, IL-6, and TNF-*α* levels (*n* = 6). * *p* < 0.05; ** *p* < 0.01; *** *p* < 0.001; **** *p* < 0.0001.

We used the autophagy inhibitor 3-methyladenine (3-MA) to clarify the relationship between ferroptosis and autophagy in a NASH mouse model. We found that 3-MA intervention ameliorated the MCD diet-induced mitochondrial volume reduction and autophagosome aggregation (Figure 7A). Notably, after the treatment with 3-MA, the levels of ROS, Fe^2+^ and MDA were reduced, while the SOD and GSH levels in liver tissues were increased compared with MCD group (Figure 7B–G). This was further confirmed by Western blot results (Figure 7H). In addition, we found that MCD diet-induced inflammation resolved after treatment with an autophagy inhibitor (Figure 7I–M). After autophagy was inhibited, ferroptosis-related proteins underwent benign changes. Taken together, MCD diet-induced NASH in mice is accompanied by autophagy, which further induces ferroptosis to promote NASH.

## 3. Discussion

NASH is mainly characterized by stem cell macrovesicular steatosis with stem cell damage and inflammation, and patients with severe disease may develop liver cirrhosis and liver cancer [14]. An MCD diet is a classic method to induce NASH in animal models. MCD diets promote the dysfunction of liver mitochondrial *β*-oxidation, thereby establishing an animal model of NASH [15]. This model is mainly used to study inflammation, oxidative stress, mitochondrial damage, and liver fibrosis related to NASH [16,17]. This model has the advantages of a short modeling time, little influence by individual differences, and good uniformity and stability of liver inflammation [18]. In this study, C57BL/6J mice were fed an MCD diet to establish a NASH model. The MCD diet lacks choline, which is involved in the synthesis of phospholipid membranes and is a vital essential nutrient for cells [19]. Therefore, PPC was selected as a positive control. Although there are currently no first-line drugs approved to treat NAFLD, PPC has been widely used by clinicians to treat patients suffering from NAFLD [20].

HE and Oil Red O staining results showed that the MCD diet caused massive lipid accumulation, round fat vacuoles, and obvious balloon-like changes in mouse hepatocytes. In addition, immunofluorescence analyses and ELISAs also confirmed the accumulation of inflammatory factors and the infiltration of inflammatory cells in the model. AP supplementation alleviated the fat accumulation and inflammation caused by the MCD diet.

Autophagy, as a homeostatic mechanism, is ubiquitous in eukaryotic cells. When the cellular environment is not conducive to normal body functions, such as damage and malnutrition, autophagy increases, and damaged organelles and proteins are removed to keep the cells in a stable state and protect them from further damage [21,22]. Functionally, autophagy plays an important role in maintaining body homeostasis, and the occurrence of many diseases is closely related to autophagy disorders [23]. Autophagy has been shown to play a key role in the development of NAFLD [24]. Autophagy is closely related to lipid metabolism in hepatocytes. Impaired autophagy in the liver can promote the storage of triglycerides in lipid droplets, leading to increased lipid droplet formation and liver steatosis. The inhibition of autophagy or deletion of autophagy genes reduces lipid droplet accumulation and protects the liver from steatosis [25]. Unlike NAFLD, excessive autophagy has been reported to increase hepatocyte inflammation and oxidative stress, exacerbating NASH [26]; this observation is consistent with our experimental results.

The p53 protein, a member of the p53 family, regulates cellular responses to various stress signals [27]. mTOR is a key regulator in the initiation of autophagy and can negatively regulate the occurrence of autophagy [28]. The p53/mTOR pathway plays an important role in the process of autophagy. After a cell is stressed, p53 is expressed in large quantities and activates mTOR, thereby upregulating the level of autophagy [29]. Beclin-1 participates in the formation of autophagic vesicles and is an important modifier in the initiation stage of autophagy [30]. LC3B is a specific marker of autophagosome formation and an important indicator of autophagic vesicle formation; therefore, it is widely used to monitor autophagy [31]. p62 is a multifunctional signaling molecule. As autophagy levels increase, p62 is continuously consumed [32]. The reduction in Beclin-1 can cause the aggregation of the autophagy substrate p62, which can form a complex with the LC3B protein and ubiquitinated proteins on the autophagosome membrane to complete the degradation process in autolysosomes [33]. In this study, we confirmed that AP may inhibit autophagy in hepatocytes by downregulating p53/mTOR pathway.

Ferroptosis involves the abnormal accumulation of iron ions and refers to a new mode of programmed cell death that leads to an imbalance in oxidation–reduction reactions by producing excessive ROS through the Fenton reaction [34]. Ferroptosis is mainly characterized by the accumulation of iron-dependent toxic lipid peroxides. Its morphological characteristics are distinguished from apoptosis, necrosis, and autophagy, mainly manifesting as decreased mitochondrial volume, increased membrane density, and decreased or absent mitochondrial cristae [35]. Our electron microscopy results showed that in mouse hepatocytes, an MCD diet caused obvious symptoms of ferroptosis, and AP reversed the effects of ferroptosis.

The liver is an important organ for iron storage and lipid metabolism. Abnormal iron metabolism and the excessive accumulation of lipid peroxides in hepatocytes can cause ferroptosis [36]. Studies have shown that intervention with ferroptosis agonists in MCD diet-induced NASH model mice aggravates liver steatosis and that treatment with ferroptosis inhibitors reduces the severity of NASH [37]. The main feature of ferroptosis is Iron-dependent lipid ROS overload. After the inactivation of GPX4, a key regulator of ferroptosis, lipid peroxides are susceptible to reduction by free Fe^2+^, producing excess ROS, which directly leads to ferroptosis [38]. GPX4 is an antioxidant enzyme that reduces lipid peroxides to normal lipids. GPX4 expression is regulated by NRF2, an endogenous antioxidant factor, which is mostly present in the cytoplasm under nonoxidative stress and is coupled to the adaptor protein KEAP1 [39]. When stimulated by ROS, it is uncoupled from KEAP1 and translocated to the nucleus. It also promotes the degradation of FTH1 and promotes ferroptosis [40]. NCOA4 (nuclear receptor coactivator 4) is a selective cargo receptor for the autophagic turnover of ferritin by lysosomes, which is a key protein in the development of autophagic ferroptosis [41].

**Figure 8:**
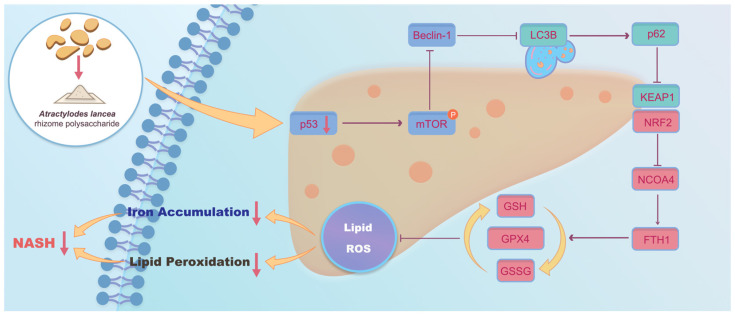
Potential mechanisms of AP in NASH mice. AP alleviates MCD diet-induced NASH by inhibiting p53/mTOR mediated autophagic ferroptosis.

Autophagy is closely related to ferroptosis, and excessive autophagy activates ferroptosis. It is not known whether autophagy exacerbates NASH through ferroptosis. The transcriptomics results showed that AP may play a role by inhibiting autophagy and ferroptosis, and the electron microscopy results also confirmed the existence of autophagy and ferroptosis. To verify the relationship between autophagy and ferroptosis in the NASH mouse model, the autophagy inhibitor 3-MA was used [42]. The results showed that the autophagy inhibitor 3-MA reversed hepatocyte ferroptosis, suggesting that hepatocyte ferroptosis is mediated by the activation of autophagy. This suggests that AP attenuates ferroptosis in hepatocytes mainly by decreasing autophagy rather than directly mediating ferroptosis through the activation of p53. The p53 signaling pathway indirectly inhibits ferroptosis in hepatocytes by inhibiting autophagy. The potential mechanism of AP in NASH mice is illustrated in Figure 8.

## 4. Materials and Methods

### 4.1. Materials and Reagents

*Atractylodes lancea* rhizome was obtained from Inner Mongolia, China. Polyene phosphatidylcholine capsules (PPCs; Cat.#H20059010) were obtained from Sanofi (Beijing, China) Pharmaceutical Co., Ltd. Assay kits for the measurement of total cholesterol (TC; Cat.#A110-1), triglycerides (TG; Cat.#A111-1), nonesterified fatty acids (NEFAs; Cat.#A042-2-1), high-density lipoprotein cholesterol (HDL-C; Cat.#A112-1-1), low-density lipoprotein cholesterol (LDL-C; Cat.#A113-1-1), alanine aminotransferase (ALT; Cat.#C009-2-1), aspartate aminotransferase (AST; Cat.#C010-2-1), superoxide dismutase (SOD; Cat.#A001-3), malondialdehyde (MDA; Cat.#A003-1), and glutathione (GSH; Cat.#A005-1) were purchased from Nanjing Jiancheng Bioengineering (NJJCBIO) Institute (Nanjing, China). ELISA kits (IL-6; Cat.#EK206; IL-1*β*; Cat.#EK201B; TNF-*α*; Cat.#EK282) were purchased from MultiSciences (Lianke) Biotechnology Corporation Limited (Hangzhou, China). Ferrous Iron (Fe^2+^; Cat.#E-BC-K773-M) Colorimetric Assay Kit was purchased from Elabscience Biotechnology Co., Ltd. (Wuhan, China). Cell Signaling Technology (Massachusetts, MA, USA) provided antibodies. We obtained HE, and oil red O staining kits from Servicebio Technology Co., Ltd. (Wuhan, China).

Trophic Animal Feed High-tech Co., Ltd. (Nantong, China), provided the L-amino acid diet, which contains 60 kcal% fat, 0.1% methionine, and no additional choline (No. TP 36225MCD). The methionine-choline-supplemented (MCS) diet, matches to the MCD diet, and includes an L-amino acid diet that has 10 kcal% fat and contains methionine and choline. This diet was also provided by Trophic Animal Feed High-tech Co., Ltd. (No. TP 36225MCS).

### 4.2. Preparation of AP

Atractylodes specimens were sliced and extracted in boiling water (1:10 volume ratio) at 100 °C for 2 h; three extractions were performed. The extract was concentrated, and 95% ethanol was added for precipitation (the final concentration of ethanol was maintained above 80%). The extract was left at 4 °C for 10 h to precipitate and then vacuum-evacuated and filtered. The precipitate was placed in a vacuum drying oven for 12 h.

### 4.3. Structural Characterization of AP

#### 4.3.1. Determination of the Molecular Weight of AP

Elution was performed using a NaCl solution (0.2 M) at a flow rate of 0.8 mL/min. A BRT105-103-101 tandem gel column (8 × 300 mm) and a differential detector (RID-20A) were used to obtain the chromatogram and retention time, draw a standard curve, obtain the molecular weight calculation formula, and calculate the molecular weight (Mp, Mw, Mw).

#### 4.3.2. Determination of the Monosaccharide Composition

Five milligrams of sample was weighed, and 2 mL of 3 M TFA was added to the sample for hydrolysis at 80 °C for 3 h. The sample was dried under nitrogen gas, vortexed and mixed with 5 mL of water; 50 µL was aspirated and added to 950 µL of deionized water, followed by centrifugation at 12,000 rpm for 5 min. The analytical conditions were as follows: column, Dionex Carbopac TM PA20 (3 × 150 mm) (Thermo, Waltham, MA, USA); mobile phase, A: H_2_O, B: 15 mM NaOH, and C:15 mM NaOH and 100 mM NaAc; flow rate, 0.3 mL/min; injection volume, 25 µL; and column temperature, 30 °C.

#### 4.3.3. Fourier Infrared Spectroscopy

AP (2 mg) and potassium bromide (200 mg) were weighed and then crushed, and potassium bromide powder was used as a blank control. The samples were scanned and recorded with an FT-IR650.

### 4.4. Establishment and Treatment of the NASH Model

Male C57BL/6J mice (5 weeks old, HFK Biochemical Technology Co., Ltd., Beijing, China) were housed at the Institute of Laboratory Animal Science, Jinan University (Guangzhou, China), following the appropriate experimental protocols (Animal Ethics No. IACUC-20220114-06). Throughout the experiment, all animals had unrestricted availability to nourishment and hydration and were housed in a 12-h light/dark cycle. The temperature was 25 °C, and the relative humidity was 50–60%.

In the first experiment, 40 mice were randomly divided into four groups according to body weight (*n* = 10 each): MCS group, MCD group, MCD+AP group, and MCD+PPC group. The mice in the MCD, MCD+AP and MCD+PPC groups were fed a methionine-choline-deficient diet for 3 weeks, and the mice in the MCS group were fed a methionine-choline-supplemented diet for 3 weeks. All mice received oral gavage daily. The MCD+AP group received AP (100 mg/kg/d), the MCD+PPC group received PPC (120 mg/kg/d), and the MCS and MCD groups received an equal volume of deionized water.

In the second experiment, 30 mice were randomly divided into three groups according to body weight (*n* = 10 each): MCS group, MCD group, and MCD+3-MA group. The mice in the MCD and MCD+3-MA groups were fed a methionine-choline-deficient diet for 3 weeks, and the mice in the MCS group were fed a methionine-choline-adequate diet for 3 weeks. The MCD+3-MA group, which received intraperitoneal injections of 3-MA (15 mg/kg/d, dissolved in DMSO; MedChemExpress, Monmouth Junction, NJ, USA).

### 4.5. Histopathological Examination

Samples of liver tissue were obtained after death and promptly preserved in 4% paraformaldehyde for histopathological analysis. The preserved tissues were encased in paraffin, sliced into 5 micron-thick sections, and subjected to HE staining for overall histological assessment. Liver tissues fixed with 4% paraformaldehyde were dropped with OCT embedding agent after dehydration and made into 10 μm frozen slices for Oil Red O (ORO) staining. To assess inflammation, immunofluorescence (IF) staining was performed using antibodies against F4/80. Fluorescently labelled samples were examined using a microscope to observe areas with discoloration. In addition, we performed DHE staining in strict adherence to the manufacturer′s instructions to assess the levels of reactive oxygen species (ROS) in liver tissues. ROS levels were measured by capturing the fluorescence intensity of DHE using a fluorescence microscope and quantified with image analysis software. To conduct thorough examinations of liver tissue at the microscopic level, we immediately stored small liver sections in a solution containing 2.5% glutaraldehyde buffered with 0.1 M phosphate (pH 7.4). Following rinses in phosphate buffer, the specimens underwent postfixation in 1% osmium tetroxide. Subsequent steps included dehydration through an incremental ethanol series and embedding within EPON resin. Thin slices, approximately 70 nm thick, were created using an ultramicrotome, placed on copper mesh grids, and then stained with uranyl acetate and lead citrate. Observations were made utilizing TEM (TECNAI G2 SPIRIT TWINE, FEI, Charlottesville, VA, USA).

### 4.6. Biochemical Analysis

The blood samples were subjected to centrifugation at a temperature of 4 °C and a speed of 3600 rpm for 15 min. The serum biomarkers ALT, AST, HDL-C, and LDL-C were then assessed using kits following the procedures of manufacturers. The liver specimens were weighed, homogenized, and subsequently centrifuged to obtain supernatants from the homogenates. Afterward, the levels of TC, TG, and NEFA in the liver homogenates were evaluated by utilizing the corresponding TC, TG, and NEFA detection kits.

### 4.7. Cytokine Analysis Using ELISAs

Parts of the liver tissues in each group were homogenized with lysis buffer to extract total protein. The homogenate was centrifuged at 12,000× *g* at 4 °C for 15 min to collect the supernatant. The levels of IL-1β, IL-6, and TNF-*α* in the tissues were measured using ELISA kits according to the manufacturer’s instructions and quantified according to the standard curve. All experiments were repeated six times and the levels of cytokines were expressed in pg/mL.

### 4.8. Detection of Oxidative Stress Indexes

Homogenization was performed using the sample homogenization buffer provided with the kit. Subsequently, the homogenate was centrifuged at 12,000 rpm for 10 min. The supernatant was collected, and the levels of SOD, MDA, GSH, and Fe^2+^ in the supernatant were measured according to the instructions of the assay kit. All experiments were repeated six times.

### 4.9. Liver Lipidomic Analysis

The lipid composition was examined with a UHPLC system connected to a high-resolution MS/MS instrument. To cover a wide range of lipid categories, we utilized both positive and negative ion modes in the mass spectrometer. Lipid identification was performed by comparing the precise mass, retention time, and MS/MS spectra with a reference lipid database. Lipid quantification was conducted by normalizing peak areas.

### 4.10. Liver Transcriptomic Analysis

Extracted RNA was evaluated for quality and quantity and analyzed for differential gene expression using the R environment, and *p* values less than 0.05 indicated differentially expressed genes (DEGs).

### 4.11. Western Blot Analysis

As described in our previous experimental approaches, we utilized RIPA lysis buffer enriched with protease and phosphatase inhibitors to extract total protein from liver tissue samples. The protein concentration was measured using a BCA protein assay kit. SDS–PAGE was used to separate protein amounts uniformly on 8% gels, which were then transferred to PVDF membranes. The specified proteins were incubated with primary antibodies at a temperature of 4 °C overnight. Following a washing process, the membranes were then incubated with secondary antibodies conjugated with HRP for 1 h at room temperature. The protein bands were observed utilizing an enhanced chemiluminescence (ECL) detection mechanism, and the intensity of the bands was measured using image analysis software. The expression levels of the target proteins were standardized by comparing them to the expression levels of the housekeeping proteins.

### 4.12. Statistical Analysis

GraphPad Prism 9.0 software was used for all the statistical computations. One-way ANOVA was used for statistical analysis in this study. The statistical results are expressed as the mean ± SD. Values of *p* < 0.05 were regarded as statistically significant.

## 5. Conclusions

In conclusion, AP alleviates MCD diet-induced NASH by inhibiting p53/mTOR mediated autophagic ferroptosis and reducing lipid accumulation and inflammatory response. These preliminary findings suggest that AP may be an ideal natural candidate for new therapies for NASH.

## Data Availability

The raw data supporting the conclusions of this article will be made available by the authors on request.
